# Effective Treatment of *Staphylococcus aureus* Intramammary Infection in a Murine Model Using the Bacteriophage Cocktail StaphLyse™

**DOI:** 10.3390/v15040887

**Published:** 2023-03-30

**Authors:** Eric Brouillette, Guillaume Millette, Suzanne Chamberland, Jean-Pierre Roy, Céline Ster, Tadele Kiros, Stephanie Hickey, Lauren Hittle, Joelle Woolston, François Malouin

**Affiliations:** 1 Département de Biologie, Faculté des Sciences, Université de Sherbrooke, Sherbrooke, QC J1K 2R1, Canada; 2 Techniques de Santé Animale, Cégep de Sherbrooke, Sherbrooke, QC J1E 4K1, Canada; 3 Sherbrooke Research and Development Centre, Agriculture and Agri-Food Canada, Sherbrooke, QC J1M 0C8, Canada; 4 Phileo by Lesaffre North America Office, 7475 West Main Street, Milwaukee, WI 53214, USA; 5 Intralytix, Inc., Columbia, MD 21046, USA

**Keywords:** *S. aureus*, intramammary infection, bovine mastitis, bacteriophage, phage therapy

## Abstract

*Staphylococcus aureus* causes intramammary infections (IMIs), which are refractory to antibiotic treatment and frequently result in chronic mastitis. IMIs are the leading cause of conventional antibiotic use in dairy farms. Phage therapy represents an alternative to antibiotics to help better manage mastitis in cows, reducing the global spread of resistance. A mouse mastitis model of *S. aureus* IMI was used to study the efficacy of a new cocktail of five lytic *S. aureus*-specific phages (StaphLyse™), administered either via the intramammary (IMAM) route or intravenously (IV). The StaphLyse™ phage cocktail was stable in milk for up to one day at 37 °C and up to one week at 4 °C. The phage cocktail was bactericidal in vitro against *S. aureus* in a dose-dependent manner. A single IMAM injection of this cocktail given 8 h after infection reduced the bacterial load in the mammary glands of lactating mice infected with *S. aureus*, and as expected, a two-dose regimen was more effective. Prophylactic use (4 h pre-challenge) of the phage cocktail was also effective, reducing *S. aureus* levels by 4 log10 CFU per gram of mammary gland. These results suggest that phage therapy may be a viable alternative to traditional antibiotics for the control of *S. aureus* IMIs.

## 1. Introduction

Intramammary infections (IMIs) and subsequent mastitis cause a decrease in milk production and degrade milk quality. Mastitis management in herds, based on hygiene and antibiotic therapy, involves the administration of intramammary or parenteral antibiotics to treat clinical cases during lactation, and to treat or prevent subclinical cases at dry-off. Antibiotic dry cow therapy (DCT) was introduced in the dairy industry in the 1970s and involves systematically treating all cows with intramammary antibiotics. DCT is still being used today to remove pre-existing IMI at dry-off and to prevent new IMI during the nonlactating period [1,2,3]. The cost associated with antibiotic treatment, the economic loss caused by having to discard milk due to the risk of antibiotic residues in milk, as well as early animal culling due to persistent mastitis, cause significant economic losses to the dairy industry worldwide [4,5].

One of the most common etiological agents of IMIs in dairy cattle is *Staphylococcus aureus*. This pathogen can cause both clinical mastitis, which is characterized by macroscopic signs of inflammation, and subclinical mastitis, which is suspected by an elevated count of somatic cells in milk and can be confirmed via bacteriological analysis of milk samples [6,7]. While most pathogens can usually be eradicated from infected quarters using antibiotics, *S. aureus* is frequently refractory to treatment and often establishes chronic IMIs [8]. Chronic *S. aureus* IMIs may last for several weeks during lactation because pathogen clearance by the host immune system is difficult [9]. Furthermore, virulence factors promoting cell invasion and biofilm formation render antibiotic therapy ineffective [10,11,12,13]. On top of it all, while some *S. aureus* strains are frequently refractory to antibiotic treatments, bovine mastitis induced by *S. aureus* is frequently difficult to cure despite the absence of antibiotic resistance [14]. The widespread use and mismanagement of antibiotic treatment in bovine mastitis contribute to the global spread of antibiotic resistance in *S. aureus*, with resistance to antimicrobials used against this pathogen steadily increasing since 2009 [15,16,17]. To better control *S. aureus* mastitis in the dairy industry and reduce the spread of resistance to antibiotics, a One Health management approach must be implemented. More effective dynamic farm management, the prudent use of antibiotics that are critically important for human medicine, and the development of alternatives to antibiotics for the control of *S. aureus* mastitis are required [15,18,19].

There is growing interest in developing bacteriophage-based products for the treatment of infections in food animals. Using bacteriophages instead of antibiotics may have several advantages, including their specific mode of action (i.e., phages will kill only the targeted pathogenic bacteria, with no deleterious impact on the normal, and often beneficial, microflora) and their excellent safety profile; phages can also be effective against bacteria that have developed resistance to all commonly used antibiotics, and the use of phages is not expected to promote the emergence of antibiotic-resistant bacteria. Bacteria can develop resistance to phages; however, resistance development may be reduced by using cocktails composed of several phages [19]. Bacteriophage (phage) therapy is still uncommon because antibiotics have been the preferred treatment for bacterial infections since the 1940s [20,21]. Lytic phages act as bactericidal agents by replicating inside the bacterial cells and killing them. Phages have been used to treat human infections in the Eastern world for decades [20], and the treatment has been found to be safe and, when phage preparations were properly selected, effective [22]. However, more phage therapy research and clinical trials are needed to determine the optimal conditions to achieve a successful treatment [23,24]. Animal studies on *S. aureus*-targeted phage therapy have shown the potential of this approach in contaminated catheters [25], subcutaneous abscesses [26], septicemia [27], bacteremia [28], sinusitis [29], and recently, in IMIs in mice [30,31,32,33,34].

Lytic phages have been isolated from cows with *S. aureus* IMI, suggesting that the conditions encountered in the mammary gland may allow phage replication [35,36,37,38]. Several factors can affect phage efficacy, including the multiplicity of infection, the dose and timing of administration, the presence of neutralizing antibodies, and the relative spectrum or specificity of the phage [39], and as a result, treatment conditions must be optimized for the treatment to be effective.

In this report, we investigated the efficacy of StaphLyse™, a cocktail of five bacteriophages lytic for *S. aureus*, in a well-characterized *S. aureus* mouse mastitis model that we have previously used to study the efficacy of novel antibiotics and vaccines for the control of *S. aureus* IMIs [40,41,42,43,44,45,46,47]. We also evaluated the effect of single and multiple phage doses, as well as the timing and route of administration on treatment outcomes.

## 2. Materials and Methods

### 2.1. S. aureus Strains

A collection of 709 *S. aureus* strains was used to determine the lytic range of the phages. This collection includes 89 methicillin-susceptible *S. aureus* (MSSA), 396 methicillin-resistant *S. aureus* (MRSA), and 2 vancomycin-intermediate *S. aureus* (VISA) strains. They were obtained from a variety of providers, including ATCC, BEI Resources, dairy farms, hospitals, and various universities. The isolates were obtained from different infections, such as wounds, mastitis, and body fluids.

*S. aureus* ATCC 49775 and ATCC 29740 were used in the subsequent experiments. *S. aureus* Rosenbach ATCC 49775 was originally isolated from a case of human furunculosis. *S. aureus* Rosenbach ATCC 29740, also known as *S. aureus* Newbould 305 [48], was originally isolated in 1958 from a cow with bovine mastitis. *S. aureus* ATCC 29740 is used as a model strain to reproducibly induce chronic mastitis in cows and is commonly used in murine models of IMIs.

### 2.2. StaphLyse™ Phage Cocktail

**Figure 1:**
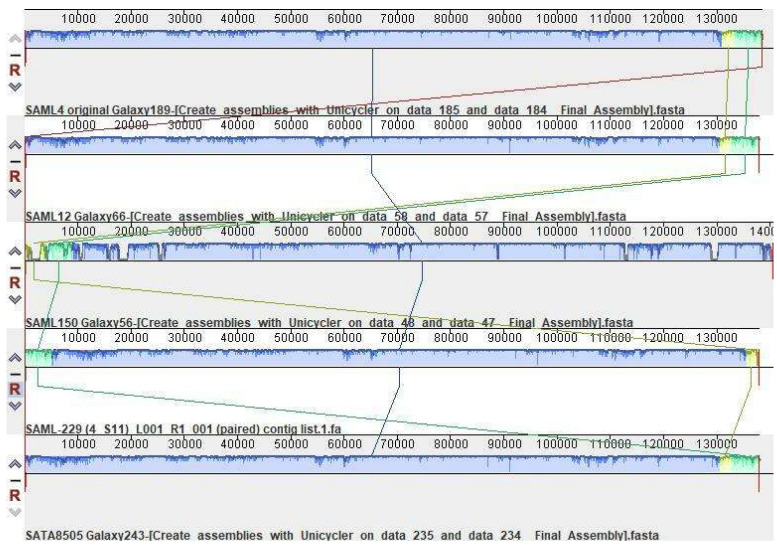
Mauve comparative genomic sequence analysis of the five *S. aureus* phages included in StaphLyse™. This method aligns conserved regions in two or more genomes. The genomes of the five StaphLyse™ phages are lined up parallel to each other. The red vertical lines indicate the genome boundaries, which were determined using the genome assembly tool (defined by long terminal repeats, which are typical of the Herelleviridae family). Colored similarity plots are shown for each genome. Different colors represent different conserved regions within the genome. These regions are referred to as locally collinear blocks (LCBs). Similar colors with connecting lines show which regions are similar between the genomes. Shading and spacing within the LCB depict variation. In this figure, there are three LCBs, shown as blue, yellow, and green, shared among the five phage genomes [50].

The StaphLyse™ phage cocktail is composed of approximately equal concentrations of five bacteriophages with lytic potency against *S. aureus* (Table 1). All five phages included in StaphLyse™ have been fully sequenced and characterized (Figure 1). These five bacteriophages belong to the Herelleviridae family, in the class Caudoviricetes; their sizes range from 137,842 bp (SAML-229) to 140,609 bp (SAML-150). These five bacteriophages meet the 40 CFR § 725.421 safety criteria established by the FDA for biological products contemplated for human therapeutic applications in terms of the absence of undesirable genes, such as genes coding for antibiotic resistance and virulence factors in their genomes [49].

### 2.3. Lytic Range of the StaphLyse™

The lytic range of the StaphLyse™ cocktail was evaluated using susceptibility testing against a collection of 709 *S. aureus* strains. Susceptibility testing was performed using the classical agar layer (Luria Bertani (LB) agar)/spot test assay essentially as described previously [51]. A force-directed graph depicting the host-killing range and the interaction network of the 5 monophages included in the StaphLyse™ cocktail was generated using the proprietary PhageSelector™ program (Intralytix, Inc., Columbia, MD, USA).

### 2.4. Stability Study

The stability of StaphLyse™ in whole milk was evaluated for 0, 1, 24 (1 day), and 168 (7 days) h. Aliquots (2.5 mL) of milk were pre-equilibrated to 37 °C or 4 °C, and 250 µL of StaphLyse™ was added to triplicate milk samples. To determine the initial phage titer, an aliquot of each sample was immediately serially ten-fold diluted in SM buffer (50 mM Tris-HCl pH7.5, 100 mM NaCl, 8 mM MgSO_4_) through the 10^−6^ dilution. Then, 10 µL triplicate aliquots of the 10^−5^ and 10^−6^ dilutions were spotted onto lawns of the titer host strain, *S. aureus* ATCC 49775. The spot test plates (LB agar) were incubated overnight at 30 °C, and plaques were counted the next day to obtain titers. The titer method was repeated at 1 h, 24 h, and 168 h (7 days).

### 2.5. In Vitro Killing of S. aureus in Whole Milk

The ability of StaphLyse™ to kill *S. aureus* ATCC 29740 in whole milk was analyzed at 0 and 1 h. The challenge *S. aureus* ATCC 29740 was grown to an OD of 0.2, and 375 µL of the bacterial suspension (~5 × 10^7^ CFU/mL) was added to each of three tubes containing 3 mL pre-warmed milk (37 °C). The control tube received 375 µL of SM buffer, one tube received 375 µL full concentration StaphLyse™ (~1 × 10^10^ PFU/mL), and 375 µL of a 1:10 dilution of StaphLyse™ (~1 × 10^9^ PFU/mL) was added to the third tube. The tubes were incubated at 37 °C for 1 h at 200 rpm. To determine the concentration of bacteria, an aliquot of each sample was serially diluted ten-fold in SM buffer through the 10^−6^ dilution method at 0 h and at 1 h. Bacterial cell counts were determined by plating 10 µL of each of the dilutions in triplicate on LB agar. The plates were incubated overnight at 37 °C, and colonies were counted the next day to obtain bacterial concentrations. The experiment was repeated on four separate days.

### 2.6. Mouse Mastitis Model

A well-characterized and validated mouse mastitis model [44] was used. Briefly, CD-1 lactating mice (Charles River Laboratories, Sherbrooke, QC, Canada) were separated from their 12–14-day-old pups, anesthetized with a mixture of ketamine/xylazine (87 and 13 mg/kg of body weight, respectively), and the teats of the fourth pair of mammary glands located from head to tail were disinfected with 70% ethanol prior to the intramammary injection of *S. aureus* ATCC 29740 (~100 CFU in 100 µL for each gland). All the injections into the mammary glands were carried out under a binocular at the experimental time points specified in the figure legends, using 1 mL tuberculin syringes and 31-gauge blunt needles. For all the experiments, each group contained four mice, with each mouse providing up to two glands (the fourth, i.e., the biggest ones).

**Figure 2:**
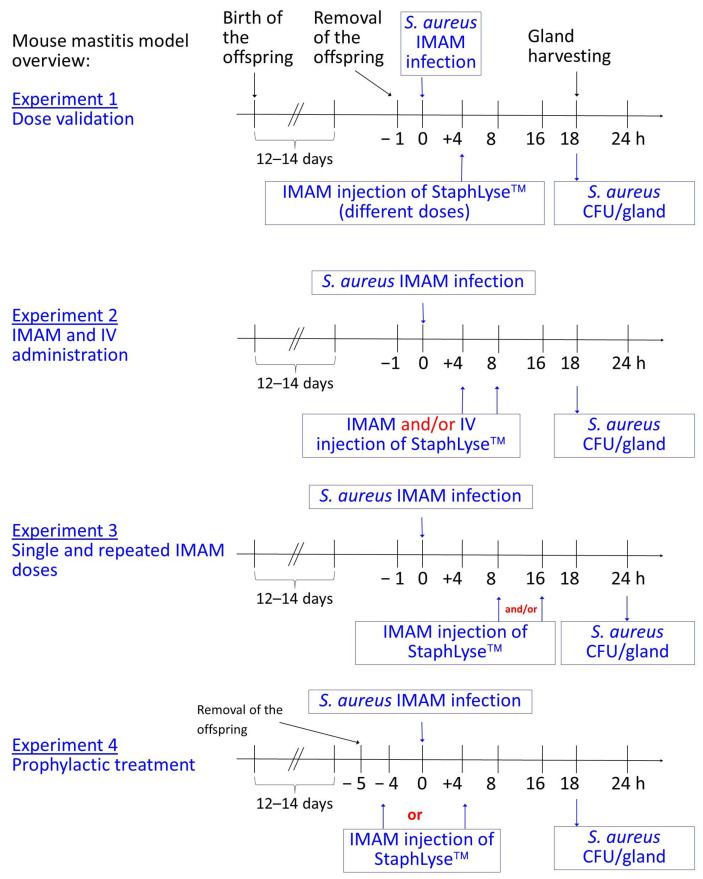
General outline of the mouse mastitis model and experimental designs. Four experimental designs were used in this study. In experiment 1, various doses of StaphLyse™ were given intramammary (IMAM). In experiment 2, StaphLyse™ was given either IMAM 4 h after *S. aureus* infection, IMAM 8 h after infection, or IMAM 4 h after infection followed by intra veinous (IV) 4 h later (at 8 h). In experiment 3, StaphLyse™ was injected via the IMAM route at 8 and/or at 16 h after *S. aureus* infection. In experiment 4, the model was used to evaluate the efficacy of prophylactic treatment. StaphLyse™ was injected via the IMAM route either 4 h prior to (−4) or 4 h after (+4) *S. aureus* infection. The results of these experiments are shown in Figures 6–9.

Figure 2 shows the general outline of the mastitis model as well as the different experimental designs that were used in this study. Four experimental designs were used. First of all, in experiment 1, the effect of different doses of StaphLyse™ was evaluated to determine the best dose to use in the following experiments. In this experiment, separate doses of StaphLyse™ (0, 10^6^, 10^7^, or 10^8^ PFU delivered in 100 µL) were administered as a single intramammary (IMAM) dose 4 h after infection. In experiment 2, we studied the effect of administration routes. StaphLyse™ or PBS were given either IMAM 4 h after infection, IMAM 8 h after infection, or IMAM 4 h after infection followed by IV (intra veinous) 4 h later (at 8 h post infection). In this experiment, each dose of StaphLyse™ contains 10^8^ PFU in a 100 µL volume. In experiment 3, we used IMAM administration, which was found to be more effective in experiment 2, and we studied the effect of administering a single dose versus two repeated doses of phage cocktail. StaphLyse™ was injected via the IMAM route at 8 and/or at 16 h after *S. aureus* infection. In experiment 4, the model was used to evaluate the efficacy of prophylactic treatment to evaluate the possibility to use StaphLyse™ as a preventive treatment for the nonlactating period in cows (DCT, as defined in the introduction section). StaphLyse™ was injected via the IMAM route either 4 h before the *S. aureus* infection (−4) or 4 h after the infection (+4).

At the end of each experiment, mice were humanely euthanized; mammary glands were harvested, weighed, and homogenized in phosphate-buffered saline (PBS); and CFUs were determined by plating serial dilutions of homogenates on tryptic soy agar (TSA). Colonies were counted after 20 h of incubation at 37 °C. In some experiments, phage titers in some of the glands were also determined using a double-layer method [52]. In brief, samples were diluted in 5 mL of molten TSA (0.7% agar) at 50 °C containing 100 µL of an overnight culture of *S. aureus* ATCC 29740. The molten agar mixture was poured onto TSA plates and allowed to set, and plates were incubated at 37 °C for 24 h. Phage plaques were counted to determine PFU/mL.

### 2.7. Statistical Analysis

Statistical analysis was performed using GraphPad Prism for Windows (version 9.3.1; GraphPad Software; San Diego, CA, USA; www.graphpad.com). For the stability of StaphLyse™ in whole milk, the data were analyzed using a two-way analysis of variance (two-way ANOVA). Sidak’s multiple-comparison test was performed to compare the stability of StaphLyse™ in milk at each temperature between 0 h and the other time points (1 h, 24 h, and 168 h). Tukey’s multiple-comparison test was performed to compare the effect of StaphLyse™ on *S. aureus* at 0 h and 1 h treatment times. In the mouse experiments, statistical significance was determined using the Kruskal–Wallis comparison test followed by Dunn’s multiple-comparison test. PFU and CFU were log10-transformed prior to statistical analysis. A *p* value < 0.05 was considered statistically significant.

## 3. Results

### 3.1. Lytic Range of the StaphLyse™ In Vitro

StaphLyse™ demonstrated lytic activity against a large collection of well-characterized *S. aureus* strains including MSSA (including *S. aureus* ATCC 29740 that was used in the mouse experiments), MRSA, and VISA strains. The cocktail lysed 92.7% of the strains tested at a titer of 2 × 10^4^ PFU/mL and 100% of these same strains when tested at a higher titer of 1 × 10^9^ PFU/mL (Table 2).

**Figure 3:**
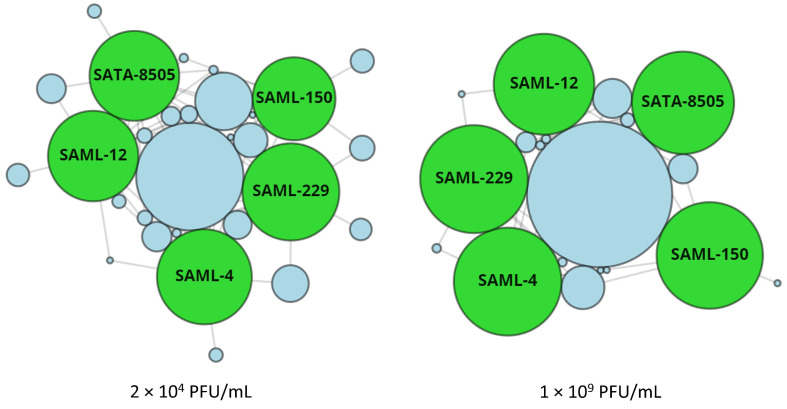
Visualization of the lytic spectrum of StaphLyse™ at two concentrations. Shown is a force-directed graph depicting the host-killing range and interaction network of the 5 monophages included in StaphLyse™ generated by the proprietary PhageSelector™ program (Intralytix, Inc., Columbia, MD, USA). Monophages are represented by green nodes, which are sized based on the total strains each kills. Blue nodes are collections of *S. aureus* strains. Lines connecting green nodes to blue nodes indicate a monophage that is capable of lysing the strains from a node at a given concentration. Blue nodes that are larger in size and are clustered closer to the center of the figure indicate that a greater number of strains are being lysed by multiple phages (i.e., redundant lytic spectrum coverage), whereas smaller nodes that are clustered closer to the edges of the figure indicate that a smaller number of strains are susceptible to fewer monophages.

Figure 3 presents the host-killing range and interaction network of the five monophages included in the StaphLyse™ cocktail. It shows that a large number of strains are being lysed by multiple phages (i.e., redundant lytic spectrum coverage).

### 3.2. Stability of StaphLyse™ and In Vitro Killing of S. aureus in Whole Milk

**Figure 4:**
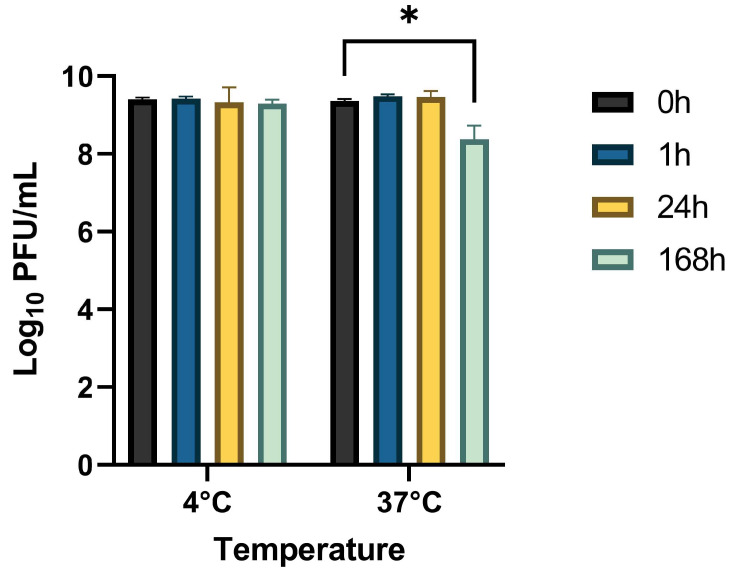
Stability of StaphLyse™ in milk at two temperatures. The average of triplicate samples is presented, and error bars represent SEM. Asterisks indicate significant differences (*p* < 0.05) for each time point compared to the respective time of 0 h for that temperature.

The stability of StaphLyse™ was evaluated after 1 h, 24 h (1 day), and 168 h (7 days) at 4 and 37 °C in milk using an initial phage concentration of ~2.5 × 10^9^ PFU/mL. Total PFU/mL counts were maintained at 4 and 37 °C for up to one day and for up to 7 days at 4 °C, while after a period of 7 days at 37 °C, a reduction of ~1.0 log10 PFU/mL was observed (Figure 4, *p* < 0.05).

**Figure 5:**
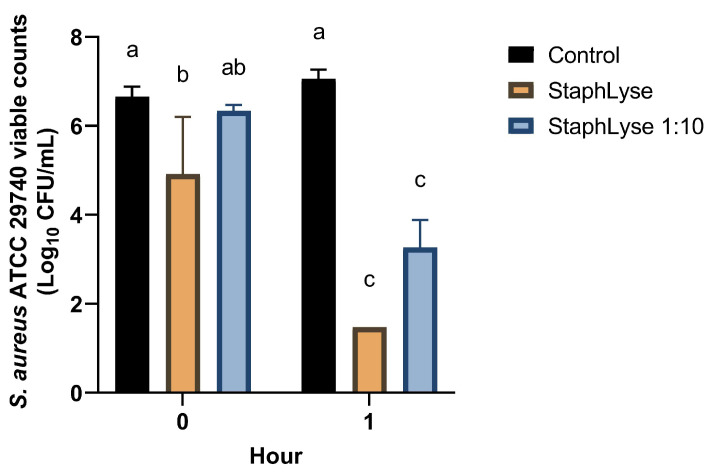
StaphLyse™ in vitro killing of *S. aureus* ATCC 29740 in whole milk. The average of four experiments is presented, and error bars represent SEM. Bars with different letters are significantly different (*p* < 0.05) from one another.

To study the in vitro killing of *S. aureus* in whole milk, two concentrations of StaphLyse™ were used: fully concentrated StaphLyse™ ~10^10^ PFU/mL and a diluted StaphLyse™ at ~10^9^ PFU/mL (StaphLyse™ 1:10). After 1 h in milk, StaphLyse™ killed 5.8 and 3.2 log10 CFU/mL of the initial *S. aureus* ATCC 29740 inoculum (6.7 log10 CFU/mL). The killing occurred quickly, as by the end of the processing at the first time point (t = 0), we already observed a reduction of 0.35 and 1.0 log10 CFU/mL at phage concentrations of 10^9^ and 10^10^ PFU/mL, respectively (Figure 5).

### 3.3. StaphLyse™ Efficacy in a Mouse Model to Treat S. aureus Intramammary Infection

**Figure 6:**
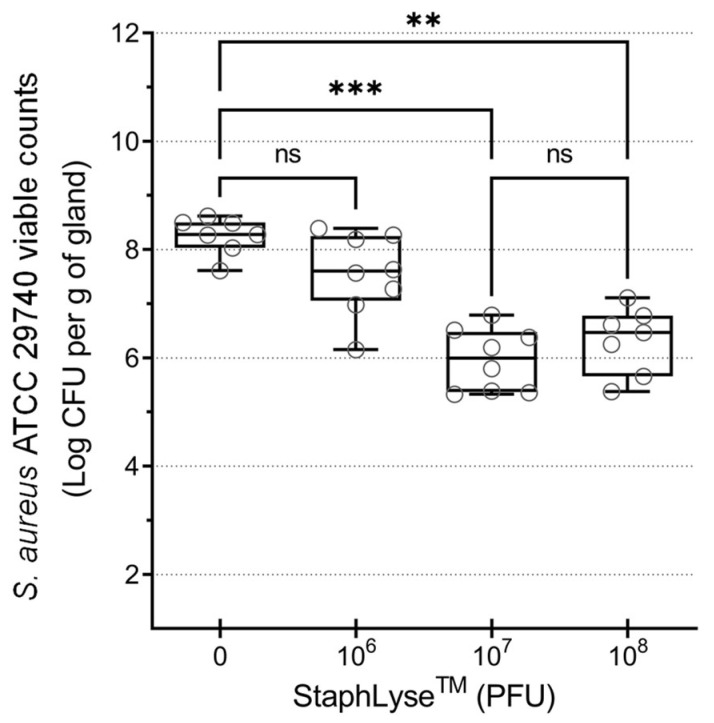
Dose-finding experiment in the mouse mastitis model of *S. aureus* infection. *S. aureus* ATCC 29740 was inoculated IMAM, at a dose of 100 CFU in a volume of 100 µL, in the 2 fourth glands in lactating CD-1 mice separated from pups 1 h prior to infection. Different doses of StaphLyse™ (in a volume of 100 µL) were administered as a single IMAM dose 4 h after infection. Mammary glands were harvested 18 h after infection, and bacterial loads into the glands were determined (CFU per gram of gland). Each group included 4 mice, with each mouse providing up to 2 glands. Each dot on the graph represents the bacterial load for each gland. The horizontal bars within the boxes indicate the median value for each group. The whiskers show the minimum and maximum values. The results were compared with the results of the placebo control group, which received PBS. Statistical significance was determined using the Kruskal–Wallis comparison test followed by Dunn’s multiple-comparison test (ns: not significant, ** *p* ˂ 0.01, *** *p* ˂ 0.001).

The efficacy of therapy with the StaphLyse™ phage cocktail was studied using a well-established mouse model of *S. aureus* IMI infection. In this model, StaphLyse™ dose ranging revealed that a single dose at phage titers of 10^7^ or 10^8^ PFU, injected via the IMAM route 4 h after infection with *S. aureus* ATCC 29740, was effective, resulting in a significant decrease in the bacterial load in the glands of infected animals, whereas a low phage titer of 10^6^ PFU was insufficient to significantly reduce the bacterial load present after 18 h of infection (Figure 6). The dose of 10^8^ PFU was selected for further experiments.

**Figure 7:**
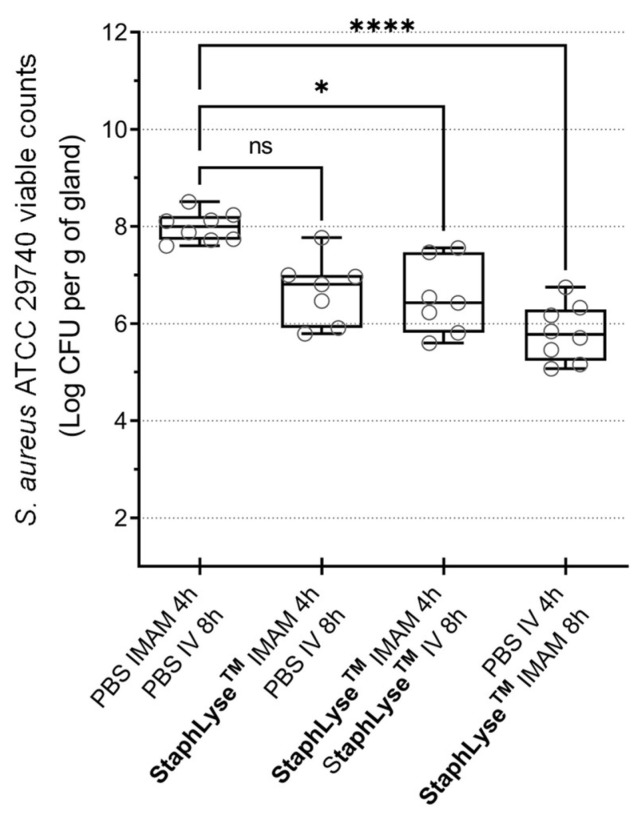
Efficacy of single and repeated doses of StaphLyse™ and effect of route of administration in the *S. aureus* mouse mastitis model. StaphLyse™ was administered via the IMAM and/or IV routes, once or twice at 4 and/or 8 h after IMAM *S. aureus* infection in lactating CD-1 mice separated from pups 1 h prior to infection. Each dose of StaphLyse™ contains 10^8^ PFU in a 100 µL volume. Each group contained 4 mice, with each mouse providing 2 glands. Glands were harvested 18 h after infection. The bacterial load for each gland is represented by a dot on the graph. The horizontal bars within the boxes indicate the median value for each group. The whiskers show the minimum and maximum values. Statistical significance was determined using the Kruskal–Wallis comparison test followed by Dunn’s multiple-comparison test (ns: not significant, * *p* = 0.02, **** *p* ˂ 0.0001).

StaphLyse™ was then administered to mice via the IMAM and/or IV routes at a dose of 10^8^ PFU once or twice at 4 and/or 8 h after IMAM *S. aureus* infection, and mammary glands were harvested 18 h after infection to determine the remaining bacterial load. A single IMAM dose of phage cocktail given 8 h after infection significantly reduced the bacterial load in the mammary glands, whereas the same dose given 4 h after infection did not generate a significant response. Furthermore, after an initial IMAM treatment 4 h after infection, an additional IV dosing of the phage cocktail 8 h later did not improve response, indicating that the IV route of administration and/or the multiplicity of infection (MOI) may not be suitable for reaching the site of infection and eliciting a response in this model (Figure 7).

**Figure 8:**
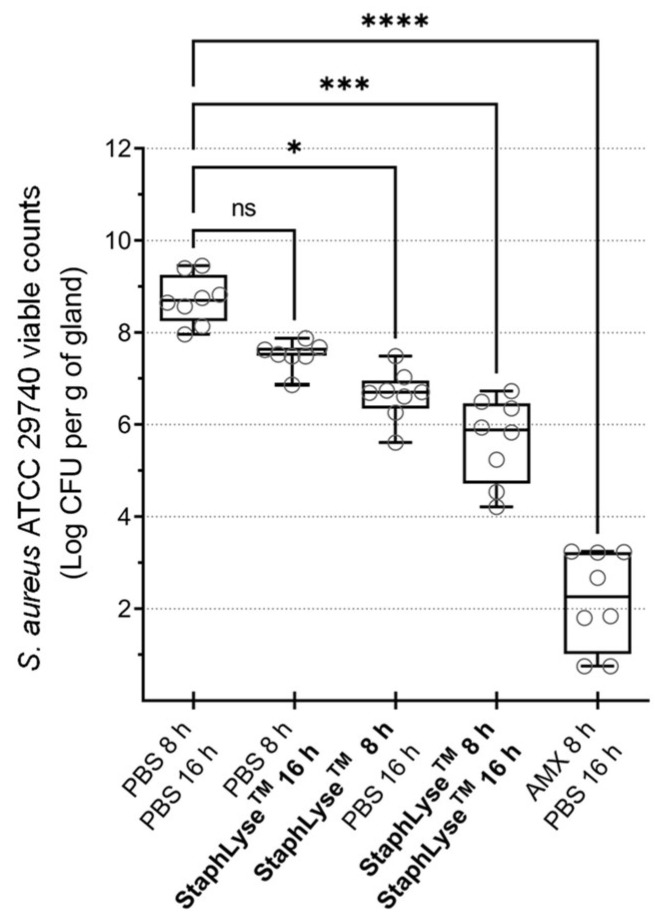
Efficacy of single and repeated doses of StaphLyse™ administered IMAM in the *S. aureus* mouse mastitis model. *S. aureus* ATCC 29740 was inoculated IMAM, at a concentration of 100 CFU in a volume of 100 µL in the 2 fourth glands, in lactating CD-1 mice separated from pups 1 h prior to infection. StaphLyse™ (10^8^ PFU in 100 µL) was administered IMAM 8 h and/or 16 h after the infection. Glands were harvested (2 per mouse) 24 h after infection. Each dot on the graph represents the bacterial load for each gland. Amoxicillin was used as a comparator and was administered IMAM at a concentration of 75 µg per gland. The horizontal bars within the boxes indicate the median value for each group. The whiskers show the minimum and maximum values. Statistical significance was determined using the Kruskal–Wallis comparison test followed by Dunn’s multiple-comparison tests (ns: not significant, * *p* = 0.02, *** *p* ˂ 0.001, **** *p* ˂ 0.0001).

We further studied the effect of single and repeated doses of StaphLyse™, using IMAM administration. We found that two IMAM doses administered at 8 and 16 h after infection was the most effective treatment regimen, significantly reducing bacterial load by 2.82 log10 CFU per gram of gland. In this experiment, amoxicillin was used as a positive control. It was administered IMAM at 8 h after the infection at a concentration of 75 µg per gland or approximately 75 µg/mL based on the volume of the gland, which is approximately 150 times higher than the MIC of amoxicillin for *S. aureus* ATCC 29740 (MIC = 0.5 µg/mL). As expected, this amoxicillin treatment resulted in a significant reduction of 4.45 log10 CFU per gram of gland (Figure 8). As an indicative measure, the number of phages in some glands was also estimated at the end of the experiment (24 h). Each dose of StaphLyse™ contains a total of 1.25 × 10^8^ PFU in a volume of 100 µL. There was an increase of 0.44 and 0.76 log10 PFU at 24 h in the sampled glands of the two groups that had only received one dose of the phage cocktail at either 8 h or 16 h, suggesting that phages remained stable in the gland or may have replicated. We recovered an equivalent amount of PFU in the gland as was initially inoculated in the sampled glands of the group that received two doses of the phage cocktail at 8 and 16 h after infection. The first phage cocktail injection may have reduced the bacterial population, and the second dose of phages may have further reduced the number of live bacteria in the gland, resulting in less phage replication. More systematic gland sampling for phage quantitation would be needed to confirm such a hypothesis.

### 3.4. Prophylactic Treatment of S. aureus Intramammary Infection Using StaphLyse™

**Figure 9:**
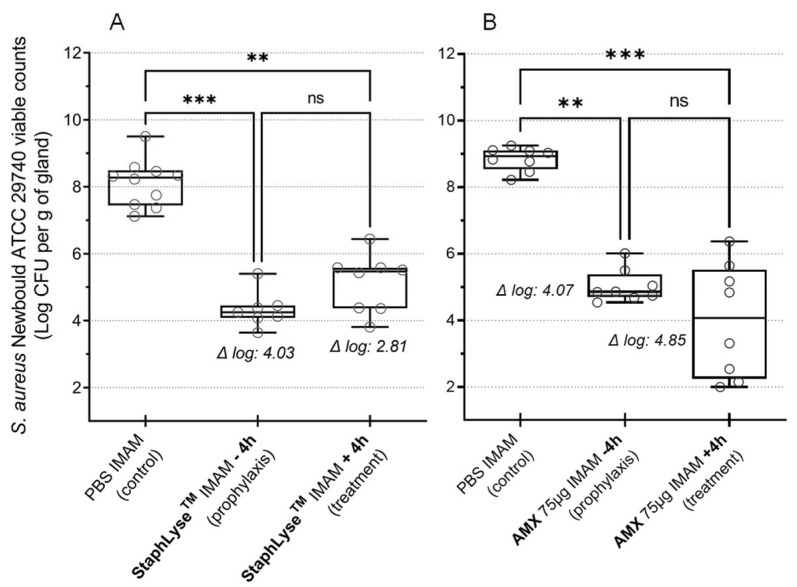
Effect of prophylactic administration of StaphLyse™ (**A**) or amoxicillin (**B**) in the *S. aureus* mouse mastitis model. *S. aureus* ATCC 29740 was inoculated IMAM, at a concentration of 100 CFU in a volume of 100 µL, in the 2 fourth glands in lactating CD-1 mice separated from pups 1 h prior to infection. StaphLyse™ (10^8^ PFU per gland) or amoxicillin (AMX, 75 µg per gland) was administered via the IMAM route at either 4 h before infection (−4 h) or 4 h after infection (+4 h). Mammary glands were harvested 18 h after infection for determination of the bacterial load (CFU per gram of gland). Each dot on the graph represents the bacterial load for each gland, whereas whiskers show the minimum and maximum values. The horizontal bars within the boxes indicate the median value for each group. Log CFU differences between controls and tests are shown in italic. Statistical significance was evaluated by the Kruskal–Wallis comparison test followed by Dunn’s multiple-comparison tests (ns: not significant, ** *p* ˂ 0.01, *** *p* ˂ 0.001).

In the dairy sector, antibiotic treatment during the cow’s nonlactating period (DCT) is performed to prevent new infection of the mammary gland when lactation resumes after calving. To test the potential of StaphLyse™ as a DCT, prophylactic treatment was evaluated in the murine model. The administration of StaphLyse™ 4 h prior to infection as well as 4 h after infection both significantly reduced the bacterial loads by 4.03 and 2.81 log10 CFU per gram of gland, respectively. Moreover, there was no significant difference in bacterial load in the glands between the two treated groups, whether the phage cocktail was administered before or after infection (Figure 9A). When the experiment was performed with amoxicillin, similar results were observed (Figure 9B). The administration of amoxicillin 4 h prior to infection as well as 4 h after infection both significantly reduced the bacterial loads by 4.07 and 4.85 log10 CFU per gram of gland, respectively. As for StaphLyse™, there was no significant difference in bacterial load in the glands between the two treated groups, whether the phage cocktail was administered before or after infection. The StaphLyse™ and amoxicillin used in prophylaxis remained active in the mammary gland for at least 4 h.

## 4. Discussion

Antibiotic resistance is currently on the rise, and it is predicted that by 2050, it will be responsible for 10 million human deaths per year, as well as USD 1 trillion loss to the global economy [53]. The overuse and misuse of antibiotics for the treatment of bovine mastitis by the dairy industry are recognized as important factors contributing to the global spread of antibiotic resistance [15,16,17]. Without available alternatives, the outright prohibition of antibiotic use in the dairy industry would result in a significant increase in production costs, due to the increased incidence of infection, and would threaten the sustainability of dairy farming [17,54]. Thus, there is an urgent need to develop alternatives to antibiotics for the treatment of bovine mastitis.

Lytic bacteriophages act as bactericidal agents and specifically infect and kill targeted bacteria. Phage therapy thus has the potential to become part of the arsenal to tackle *S. aureus* IMIs and significantly reduce the use of antibiotics by the dairy industry [55]. Phage-based products are currently used in the food safety and processing industry; however, none are yet available for administration to food animals during production [56].

In this study, we investigated the efficacy of a new phage cocktail named StaphLyse™ for the control and prevention of *S. aureus* mastitis. This phage cocktail is composed of five fully sequenced and characterized lytic phages specifically targeting *S. aureus*. We showed that the in vitro lytic activity of the cocktail was dose-dependent and that it lysed 92.7% and 100% of the strains tested (*n* = 709) at a titer of 2 × 10^4^ and 1 × 10^9^ PFU/mL, respectively (Table 2 and Figure 3). The combination of the five phages present in StaphLyse™ appears to cover a much broader range of *S. aureus* strains than that previously reported by Geng et al. for a phage cocktail composed of only two phages, one from the Myoviridae family (vBSM-A1) and the other from the Podoviridae family (vBSP-A2), with vBSM-A1 lysing 85% and vBSP-A2 lysing 59% of 23 *S. aureus* strains tested [31]. This suggests that combining more than two phages in a cocktail may be desirable to ensure efficacy against a broader spectrum of *S. aureus* strains and perhaps avoid treatment failure; however, competitive interference between different phages has been shown in *E. coli* and must be kept in mind when developing such cocktails [57]. Combining multiple phages in a therapeutic cocktail may also reduce the likelihood of resistance development [19].

A mouse mastitis model was used to study the in vivo efficacy of the phage cocktail against *S. aureus* IMI. This model is currently used to evaluate the efficacy of novel treatments for mastitis, and we have previously characterized clinical symptoms, pathological conditions, and levels of cytokines and shown that they are similar to that of a naturally occurring infection in mice [44]. Using mouse mastitis models allows researchers to test new alternatives to antibiotics in well-controlled settings and at a scale that is appropriate for the evaluation of emerging therapies. Without a doubt, mice cannot completely replace cows for all research purposes. There are differences between mouse and bovine mammary glands, and caution is needed when extrapolating mouse data to cows. However, this model clearly allows for an appreciation of the potential of novel therapies and provides further justification for the significant investment required to move forward and test such therapies in infected cows, which can only be carried out in a biosafety level 2 barn and with larger quantities of phage cocktail.

In this study, we demonstrated that the phage cocktail StaphLyse™ was effective in controlling *S. aureus* IMI in mice. A single IMAM injection of this phage cocktail given 8 h after infection via the IMAM route reduced the bacterial load in mammary glands of lactating mice infected with *S. aureus* (reduction of 2 to 2.2 log10 CFU per gram of gland, as observed in Figure 7 and Figure 8). As expected, a double-dose regimen was more effective, with a reduction of 2.82 log10 CFU per gram of gland (Figure 8). We also showed that the IV administration of phages, following an initial IMAM administration, did not improve the effectiveness of the initial IMAM treatment (Figure 7).

The pharmacodynamics and pharmacokinetics of a phage cocktail are undoubtedly different from that of traditional small-molecule antibiotics. Bacteriophages can not only kill bacteria, but they can also multiply in the environment. As a result, their pharmacology is influenced by both their direct interactions with bacterial cells as well as their capacity to penetrate tissues and reach infection sites [58]. It was shown that the tight junctions between the epithelial cells constituting the inner lining of the alveolar structure of the mammary gland are affected by neutrophil diapedesis occurring in response to inflammation caused by infection [59]. During an IMI in a cow, neutrophils migrate from the blood to the infection site within 2 to 4 h, reaching a peak in the milk between 10 and 16 h, as cytokines are released [60]. Phages injected directly into the blood may gain access to the alveolar lumen as the inflammatory response increases over time. It is also known that the inflammatory response allows blood components to enter the milk (such as serum albumin and antibodies) and vice versa (lactose from the milk leaking into the blood) as the permeability of the mammary epithelium increases [61]. This inflammatory state is typical of IMI-induced bovine mastitis, and it may have an effect on the efficacy of phage therapy administered in cows via IV injections. However, using the mouse mastitis model, Iwano et al. showed that only 1% of phages reached mammary glands and were rapidly eliminated when administered via the IV route [32].

The IMAM administration of a phage cocktail may be more practical to implement for the treatment of cows because phages can be selectively injected into the infected mammary gland quarter, directly at the site of infection. Furthermore, contrary to what was previously found for other phages in the presence of raw milk or milk whey proteins in in vitro assays [62,63], our in vitro results showed that milk did not interfere with or neutralize the lytic effect of the StaphLyse™ phage cocktail (Figure 5), suggesting that IMAM administration may be appropriate.

Finally, our study showed that a cocktail of lytic phages can be used prophylactically to control *S. aureus* mastitis in mice (Figure 9). This is an exciting finding because, if proven similarly effective in cows, phage prophylactic therapy could replace prophylactic DCT, and significantly reduce the number of antibiotics used in dairy farms.

## 5. Conclusions

The present study suggests that a cocktail of lytic phages could be a safe and effective alternative to antibiotics for the treatment or prevention of IMIs caused by *S. aureus*. The StaphLyse™ phage cocktail was stable in milk at 37 °C for up to one day and for up to one week at 4 °C. The phage cocktail was bactericidal in vitro in a dose-dependent manner against *S. aureus*. A single IMAM injection of this phage cocktail given 8 h after infection via the IMAM route reduced the bacterial load in the mammary glands of lactating mice infected with *S. aureus*, and as expected, a two-dose regimen was more effective. The prophylactic use of the phage cocktail was also effective. These results support further research to determine the efficacy of the phage cocktail StaphLyse™ in dairy cows as an alternative to traditional antibiotics therapy to control *S. aureus* IMIs.

## 6. Patents

The phages contained in StaphLyse™ and the StaphLyse™ formulation are the subject of several issued and pending patent applications, including US patent #7,745,194.

## Figures and Tables

**Table 1:** Lytic spectrum of individual *S. aureus* monophages and cumulative lytic spectrum of the StaphLyse™ phage cocktail at two concentrations.

Monophage and Cocktail	GenBank Number	Lysis of *Staphylococcus aureus* Strains ^1^					
		2 × 10^4^ PFU/mL			1 × 10^9^ PFU/mL		
		*n*	%	Unique Kill ^2^	*n*	%	Unique Kill ^2^
SAML-4	OP352121	552	77.9	5	700	98.7	0
SAML-12	OP352122	501	70.7	14	608	85.8 ^3^	0
SAML-150	OP352123	423	59.7	15	683	96.3	1
SAML-229	OP352124	580	81.8	12	700	98.7	0
SATA-8505	OQ594774	495	69.78	4	631	89.0 ^3^	0
StaphLyse™	-	657	92.7	-	709	100	-

^1^ The *S. aureus* strain collection (709 strains) used in this study includes both methicillin-sensitive and -resistant strains. ^2^ The column “unique kill” presents the number of strains that are only lysed by the monophage. ^3^ A subset of strains (i.e., those not susceptible at 2 × 10^4^ PFU/mL) were tested for SAML-12 and SATA-8505 at the higher dose (1 × 10^9^ PFU/mL); the percent kill assumes that isolates susceptible at the lower dose are also susceptible at 1 × 10^9^ PFU/mL.

**Table 2:** Lytic range of StaphLyse™ against *S. aureus*.

Phenotype ^1^	Number of Strains Tested	Lysis by StaphLyse™			
		2 × 10^4^ PFU/mL		1 × 10^9^ PFU/mL	
		*n*	%	*n*	%
MSSA **^2^**	89	76	84.4	89	100
MRSA	396	376	94.9	396	100
VISA **^3^**	2	1	50.0	2	100
Others	222	204	91.9	222	100
Total	709	657	92.7	709	100

^1^ MSSA, methicillin-susceptible *S. aureus*; MRSA, methicillin-resistant *S. aureus*; VISA, vancomycin-intermediate *S. aureus*; Others, isolates for which methicillin or vancomycin susceptibility was not determined. ^2^ StaphLyse™ was effective against *S. aureus* ATCC 29740, which was used in the mouse experiments. ^3^ VISA strains were also resistant to methicillin.

## Data Availability

All data, generated or analyzed, and materials during this study are included in this published article.
